# Clinicopathological characteristics and prognosis of microinvasive breast cancer: A population‐based analysis

**DOI:** 10.1002/cam4.4839

**Published:** 2022-05-22

**Authors:** Hangcheng Xu, Yiqun Han, Yun Wu, Yan Wang, Jiayu Wang, Binghe Xu

**Affiliations:** ^1^ Department of Medical Oncology, National Cancer Center/National Clinical Research Center for Cancer/Cancer Hospital Chinese Academy of Medical Sciences and Peking Union Medical College Beijing China

**Keywords:** breast cancer, clinical features, microinvasive carcinoma, molecular subtypes, nomogram, prognosis

## Abstract

**Objectives:**

Microinvasive breast cancer (MIBC) is a special type of breast cancer with a relatively low prevalence, of which the understanding remains controversial. In this article, we aimed to clarify the clinicopathological characteristics and prognosis of MIBC in the setting of different molecular subtypes and give feasible suggestions on clinical practice in MIBC.

**Methods:**

This study utilized the data from Surveillance, Epidemiology, and End Results (SEER) database. Patients were divided into subgroups based on the molecular subtypes, of which the clinicopathological characteristics were further undergone comparative analyses. Kaplan–Meier method and Cox proportional hazard regression analysis were employed to determine the prognosis of the subtypes, and to explore the prognostic factors. Patients were randomly assigned in a 7:3 ratio to the training and validation cohorts. The independent risk variables were then adopted to generate a nomogram to predict the 3‐ and 5‐year survival probability.

**Results:**

A total of 4301 MIBC patients between 2010 and 2016 were obtained from the SEER database, which were subsequently separated into HR+/HER2‐ (*n* = 2598), HR+/HER2+ (*n* = 723), HR‐/HER2+ (*n* = 633), and HR‐/HER2‐ (*n* = 347) groups. The HR+/HER2+ group showed the best overall survival (OS) (81.28 months, 95% CI 80.45–82.11) compared with other groups (*p* = 0.0089). The application of radiotherapy in HR+/HER2‐ and HR+/HER2+ MIBC patients brought out additional survival benefit compared with those without radiotherapy (*p* < 0.0001 and *p* = 0.024, respectively). The prognosis among four subgroups with or without chemotherapy showed no statistical difference. Based on the curated nomogram, the high‐score group exhibited a better OS compared with patients from the low‐score group.

**Conclusions:**

Profound heterogeneity was detected among different molecular subtypes in MIBC patients, of which HR+/HER2+ subtype presented the best prognosis. For HR‐positive MIBC patients, increasing survival benefits could be retrieved from radiotherapy. Chemotherapy was not recommended for patients with MIBC. Individual‐based protocols were introduced based on the nomogram which warranted further validation.

## Graphical abstract

Profound heterogeneity was detected among different molecular subtypes in MIBC patients, of which HR+/HER2+ subtype presented the best prognosis. For HR‐positive MIBC patients, increasing survival benefits could be retrieved from radiotherapy. Chemotherapy was not recommended patients with MIBC. Individual‐based protocols was introduced based on the nomogram which warrants further validation.

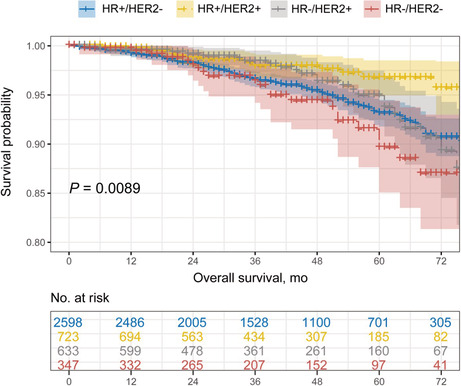

## 1 INTRODUCTION

The latest data from the GLOBALCAN 2020 indicate that breast cancer has become the most prevailing cancer worldwide, with nearly 2.3 million new cases in 2020.^1^ Thereinto, microinvasive breast cancer (MIBC) is a rare entity, accounting for only 0.68–3.4% of all breast cancer cases.^2^, ^3^ The concept of MIBC was firstly introduced in the fifth edition of the American Joint Committee on Cancer (AJCC) Cancer Staging Manual and was formally recognized as T1mic since 1997,^4^ which is defined as invasive tumor measuring no more than 1 mm in greatest dimension until now.^5^ Of note, microinvasion usually occurs in the setting of ductal carcinoma in situ (DCIS), namely DCIS with microinvasion (DCISM).^6^ With the advancement of breast cancer screening and pathology, the detection rate of MIBC increased. However, current research regarding the prognosis and clinical management of MIBC is still deficient due to its scarcity.

It is acknowledged that molecular subtype is now broadly used to guide treatment and predict the clinical outcome of breast cancer.^7^, ^8^ Several studies noticed it was significant to take the receptor status of hormone receptor (HR) and human epidermal growth factor receptor 2 (HER2) into consideration to fully elucidate MIBC.^9^, ^10^, ^11^, ^12^ It was reported that the distribution of molecular subtypes differed among DCIS, DCISM, and invasive ductal carcinoma (IDC) entity,^12^ of which the HER2 positivity and HR negativity were associated with higher nuclear grade and poorer survival in MIBC, respectively.^10^, ^13^ Most prior research mainly focused on comparing the biological behavior and prognosis of DCIS and DCISM.^14^, ^15^, ^16^ However, the prognosis data is lacking with respect to different molecular subtypes in MIBC. Except for prognosis, treatment for MIBC is another topical issue. Current management for early breast cancer includes surgery, radiotherapy, and systemic therapy.^17^ At present, controversy remains about how to implement tailored treatment for patient with MIBC to avoid both undertreatment and overtreatment.^2^ Besides, the nodal involvement is relatively rare within the MIBC entity. The majority of the previous studies reported the nodal metastasis rate was less than 10%. And the disease burden of the lymph node was generally limited to an isolated tumor cell or micrometastasis (N1mi).^18^ Macrometastasis and N0/N1mi tended to have different prognosis and clinical management as shown in some studies.^19^, ^20^ Till now, literature is sparse which focuses on the MIBC patients with N0/N1mi.

To conclude, further research on the above issues is needed. Therefore, we conducted this large‐scale population‐based analysis, attempting to clarify the clinicopathological characteristics and prognosis of MIBC. It was worth noticing that classified discussion and survival comparison was expounded in terms of different molecular subtypes, which was expected to fill the research gap in this area.

## 2 METHODS

### 2.1 Data source

Data on patients with breast cancer were extracted from the Surveillance, Epidemiology, and End Results (SEER) database (https://seer.cancer.gov/), which is a population‐based database that collects data from 18 cancer registries in the United States.^21^ The SEER database began collecting information on the molecular subtype since 2010, thus this analysis utilized the data spanned from 2010 to 2016. The cases with confirmed pathology diagnosis were included and we excluded the patients without complete population demographics and clinicopathological information. For this investigation, the information including age at diagnosis, sex, ethnicity, tumor laterality, histologic type, grade, molecular subtype, nodal status, surgical performance, radiation treatment, chemotherapeutic delivery, and survival data were collected for each patient.

### 2.2 Study variables

According to the AJCC Cancer Staging Manual (7th), T1mic was defined as extension beyond the basement membrane ≤1 mm in greatest dimension while T1a referred to that >1 mm but ≤5 mm in greatest dimension. N0 indicated no regional lymph node metastases while N1mi meant micrometastases (larger than 0.2 mm, but none larger than 2.0 mm). Molecular subtypes were classified into the four following varieties based on the SEER terminology: HR‐positive/HER2‐negative, HR‐positive/HER2‐positive, HR‐negative/HER2‐positive, and HR‐negative/HER2‐negative. HR consists of estrogen receptor (ER) and progesterone receptor (PgR). ER‐ and/or PgR‐positive was considered as HR‐positive while ER‐ and PgR‐negative were defined as HR‐negative. The SEER database only recorded features of the invasive component when in situ and invasive components were present simultaneously in a tumor. The primary end point in this study was overall survival (OS) which was defined as the interval from the diagnosis to the death caused by any reasons or the last follow‐up.

### 2.3 Statistical analysis

The clinicopathological characteristics were presented as counts and percentages. We used χ^2^ tests and Fishers' exact probability tests to compare the differences between categorical data. As for continuous data, *t*‐test, or Wilcoxon rank test was performed based on the data distribution. The survival data were analyzed using Kaplan–Meier method and log‐rank test was used to determine the differences. The univariate, as well as multivariate Cox proportional hazards regression model, was employed to explore the prognostic factors, of which the results were reported as hazard ratios (HR) with 95% confidence intervals (95% CI). To further eliminate objective differences in baseline characteristics between different cohorts, we performed propensity score matching (PSM) analysis. In addition, the enrolled patients were randomly divided into the training cohorts and the validation cohorts in a ratio of 7:3, with 3010 and 1291 patients in each group. Nomogram predicting 3‐ and 5‐year survival of MIBC patients was constructed and was internally validated for discrimination and calibration. The optimal cut point of the nomogram score was identified by the maximally selected rank statistics. A two‐sided *p* < 0.05 was considered statistically significant. All statistical analyses were performed by SPSS (version 26.0; IBM Corp.) and R software (version3.6.4; https://www.r‐project.org/).

## 3 RESULTS

### 3.1 Patient characteristics

A total of 4301 MIBC patients who met the selection criteria were involved in this study. The median age at diagnosis was 59.0 years. The population demographics and clinicopathological characteristics were presented in Table 1.

**TABLE 1:** Baseline characteristics

Characteristics	T1mic (*N* = 4301)	
	No.	Percent (%)
Age, years	59.0	
Age group, years		
<50	924	21.5
50–69	2489	57.9
≥70	888	20.6
Sex		
Female	4282	99.6
Male	19	0.4
Ethnicity		
White	3195	74.3
Black	505	18.3
Others	565	13.1
Unknown	36	0.8
Laterality		
Left	2159	50.2
Right	2142	49.8
Histologic type		
Ductal	3492	81.2
Lobular	245	5.7
Others	564	13.1
Grade		
Grade1	940	21.9
Grade2	1330	30.9
Grade3	790	18.4
Undifferentiated	31	0.7
Unknown	1210	28.1
Subtype		
HR+/HER2‐	2598	60.4
HR+/HER2+	723	16.8
HR‐/HER2+	633	14.7
HR‐/HER2‐	347	8.1
ER		
Positive	3266	75.9
Negative	1030	23.9
Borderline/Unknown	5	0.1
PgR		
Positive	2650	61.6
Negative	1625	37.8
Borderline/Unknown	26	0.6
HER2		
Positive	1356	31.5
Negative	2945	68.5
N		
N0/N1mi	4095	95.2
N1	170	4.0
N2	14	0.3
N3	9	0.2
NX	13	0.3
Surgery		
Yes	4227	98.3
No/Unknown	74	1.7
Radiotherapy		
Yes	1930	44.9
No/Unknown	2371	55.1
Chemotherapy		
Yes	439	10.2
No/Unknown	3862	89.8

Abbreviations: HR, hormone receptor; HER2, human epidermal growth factor receptor 2; ER, estrogen receptor; PgR, progesterone receptor.

Patients were divided into four groups based on the molecular subtypes, comprising HR+/HER2‐ (*n* = 2598), HR+/HER2+ (*n* = 723), HR‐/HER2+ (*n* = 633), and HR‐/HER2‐ (*n* = 347). Substantial differences were detected and summarized in Table 2. The median age at diagnosis was different in these four groups (*p* < 0.0001), of which the age in HER2‐negative groups was 61.0 years, relatively older than that of HER2‐positive groups (both were 55.0 years). As for the clinicopathological characteristics, HR+/HER2‐ group tended to have an increasing rate of special histologic subtype (15.3%), a lower tumor histologic grade (grade 1, 33.1%), and a less incidence of nodal involvement (N0/N1mi, 96.2%). Regarding the treatment, almost all patients underwent surgery, whereas the choice of radiotherapy and chemotherapy was divergent (*p* = 0.001 and *p* < 0.0001, respectively). The HER2‐positive patients had a higher rate of receiving chemotherapy and were less accessible to radiotherapy (37.6%).

**TABLE 2:** Baseline characteristics of different molecular subtypes

Characteristics	HR+/HER2− (*N* = 2598)		HR+/HER2+ (*N* = 723)		HR‐/HER2+ (*N* = 633)		HR‐/HER2− (*N* = 347)		*p* Value
	No.	Percent (%)	No.	Percent (%)	No.	Percent (%)	No.	Percent (%)	
Age, years	61.0		55.0		55.0		61.0		<0.0001
Age group, years									<0.0001
<50	484	18.6	207	28.6	168	26.5	65	18.7	
50–69	1477	56.9	423	58.5	387	61.1	202	58.2	
≥70	637	24.5	93	12.9	78	12.3	80	23.1	
Sex									0.058
Female	2581	99.3	721	99.7	633	100.0	347	100.0	
Male	17	0.7	2	0.3	0	0.0	0	0.0	
Ethnicity									0.029
White	1922	74.7	530	74.2	474	75.2	269	77.5	
Black	326	12.7	76	10.6	59	9.4	44	12.7	
Others	326	12.7	108	15.1	97	15.4	34	9.8	
Laterality									0.554
Left	1293	49.8	350	48.4	340	53.7	176	50.7	
Right	1304	50.2	373	51.6	293	46.3	171	49.3	
Others	1	0.0	0	0.0	0	0.0	0	0.0	
Histologic type									<0.0001
Ductal	1979	76.2	640	88.5	567	89.6	306	88.2	
Lobular	221	8.5	11	1.5	6	0.9	7	2.0	
Others	398	15.3	72	10.0	60	9.5	34	9.8	
Grade									<0.0001
Grade1	860	33.1	49	6.8	13	2.1	18	5.2	
Grade2	847	32.6	246	34.0	154	24.3	83	23.9	
Grade3	248	9.5	170	23.5	242	38.2	130	37.5	
Undifferentiated	10	0.4	8	1.1	8	1.3	5	1.4	
Unknown	633	24.4	250	34.6	216	34.1	111	32.0	
N									0.011
N0/N1mi	2498	96.2	676	93.5	598	94.5	323	93.1	
N1	81	3.1	39	5.4	29	4.6	21	6.1	
N2	4	0.2	3	0.4	5	0.8	2	0.6	
N3	5	0.2	3	0.4	0	0.0	1	0.3	
NX	10	0.4	2	0.3	1	0.2	0	0.0	
Surgery									0.586
Yes	2550	98.2	714	98.8	622	98.3	341	98.3	
No	48	1.8	9	1.2	11	1.7	6	1.7	
Radiotherapy									0.001
Yes	1204	46.3	320	44.3	238	37.6	168	48.4	
No/Unknown	1394	53.7	403	55.7	395	62.4	179	51.6	
Chemotherapy									<0.0001
Yes	149	5.7	127	17.6	115	18.2	48	13.8	
No/Unknown	2449	94.3	596	82.4	518	81.8	299	86.2	

Abbreviations: HR, hormone receptor; HER2, human epidermal growth factor receptor 2.

### 3.2 Prognostic profiles

**FIGURE 1:**
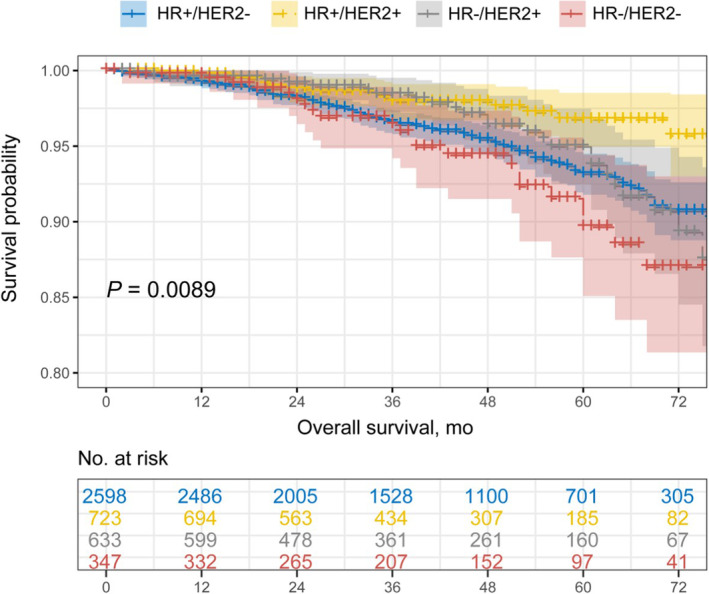
The comparison of survival outcomes of MIBC patients among four molecular subtypes.

**FIGURE 2:**
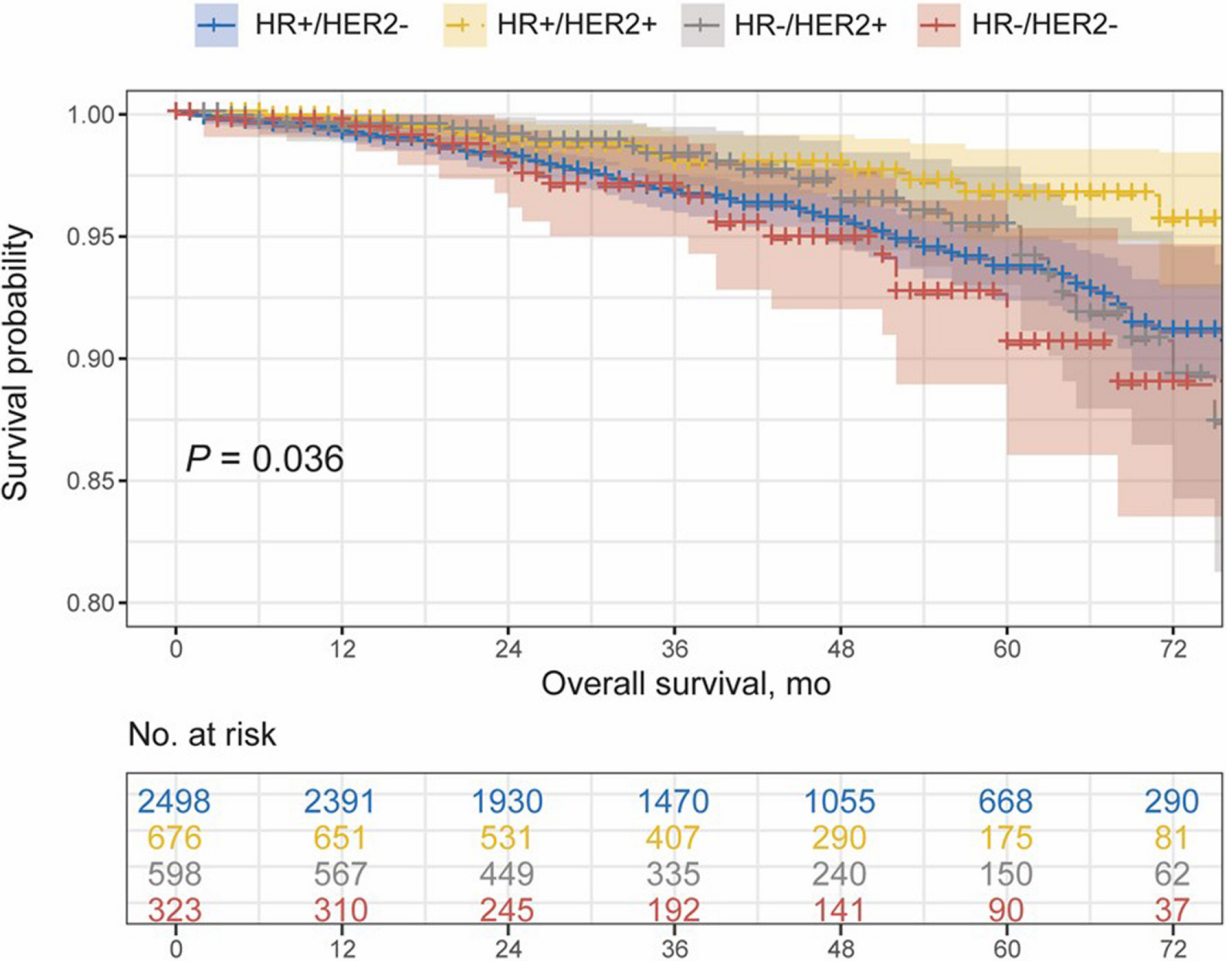
The comparison of survival outcomes of MIBC patients with N0/N1mi among four molecular subtypes.

An obvious difference in overall prognosis was found among four molecular subtypes (*p* = 0.0089) (Figure 1). The detailed survival data was demonstrated in **Table** **S1**. The median OS (mOS) of HR+/HER2‐, HR+/HER2+, HR‐/HER2+, and HR‐/HER2‐ groups was 79.32 months (95% CI, 78.69–79.95), 81.28 months (95% CI, 80.45–82.11), 79.72 months (95% CI, 78.49–80.95), and 78.19 months (95% CI, 76.29–80.10), respectively. HR+/HER2+ group had a better prognosis compared with HR+/HER2‐ group (81.28 months vs. 79.32 months, *p* = 0.003) and HR‐/HER2‐ group (81.28 months vs. 78.19 months, *p* = 0.001). No apparent difference was detected among comparisons between HR‐/HER2‐ and HR + HER2‐ or HR‐HER2+ subgroup patients. Further analysis was carried out according to the ER, PgR, HR, and HER2 status (**Figure** **S1**). Regardless of HR status, a superior prognosis was identified in HER2+ group compared with HER2‐ group (*p* = 0.0069). There was no significant discrepancy in ER, PgR, or HR intra‐group. A notable discrepancy was also detected in the prognosis of N0/N1mi proportion (*p* = 0.036) (Figure 2), of which HR+/HER2+ group had the best prognosis among the entire groups, while HR‐/HER2‐ group had the worst prognosis when compared with HR+/HER2‐ or HR+/HER2+ group (*p* = 0.019 and *p* < 0.001, respectively) (**Table** **S2**).

Subsequently, the OS was compared between T1mic and T1a patients. The prognosis was comparable between these two groups (**Figure** **S2**). The same results were obtained when further compared the OS between different subgroup divided based on the molecular subtype (**Figure** **S3**). In addition, PSM analysis was performed in a 1:2 ratio to achieve a maximum control of the confounding factors at baseline. Still, no statistical significance was detected in OS neither in the overall analysis (**Figure** **S4**) nor in the subgroup analysis (**Figure** **S5**).

Then, the effect of different treatment regimen (radiotherapy or chemotherapy) on prognosis was explored. With respect to the radiotherapy, a profound heterogeneity was identified in HR+/HER2‐ and HR+/HER2+ molecular subtypes (*p* < 0.0001 and *p* = 0.024, respectively) (**Figure** **S6**). As for the delivery of chemotherapy, no significant difference was detected in four molecular subtypes (**Figure** **S7**). In T1micN0/N1mi group, the above‐mentioned outcome remained consistent (**Figure** **S8‐9**). Besides, no divergent survival benefits could be provided by therapeutic options, including surgery, radiotherapy, and chemotherapy, for the patients between T1mic and T1a groups (**Figure** **S10**).

Finally, prognostic factors for MIBC patients were explored and demonstrated in Table 3. Results derived from the univariate regression analysis indicated that factors including age group (*p* < 0.0001), ethnicity (*p* = 0.002), molecular subtype (*p* = 0.011), surgery (*p* < 0.0001), and radiotherapy (*p* = 0.001) might have a significant impact on survival. Further analysis revealed that molecular subtype might not be an independent indicator for the prognosis of MIBC patients.

**TABLE 3:** The univariate and multivariate regression analysis of T1mic patients

Characteristics	Univariate		Multivariate	
	Hazard ratio (95%CI)	*p* value	Hazard ratio (95%CI)	*p* value
Age group, years		<0.0001		<0.0001
<50	Reference		Reference	
50–69	1.33 (0.80–2.22)	0.267	1.34 (0.81–2.23)	0.261
≥70	5.51 (3.37–9.00)	<0.0001	5.10 (3.10–8.39)	<0.0001
Sex		0.723		/
Female	Reference		/	
Male	1.43 (0.20–10.19)	0.723	/	/
Ethnicity		0.002		0.007
White	Reference		Reference	
Black	1.61 (1.11–2.33)	0.012	1.67 (1.15–2.43)	0.007
Others	0.55 (0.31–0.97)	0.038	0.68 (0.39–1.21)	0.190
Laterality		0.626		/
Left	Reference		/	
Right	0.93 (0.69–1.24)	0.628	/	/
Histologic type		0.596		/
DC	Reference		/	
LC	0.94 (0.49–1.79)	0.859	/	/
Others	1.22 (0.82–1.80)	0.328	/	/
Grade		0.887		/
Grade1	Reference		/	
Grade2	1.11 (0.73–1.68)	0.626	/	/
Grade3	1.24 (0.79–1.96)	0.347	/	/
Undifferentiated	1.46 (0.35–6.07)	0.602	/	/
Unknown	1.18 (0.77–1.81)	0.439	/	/
Subtype		0.011		0.082
HR+/HER2‐	Reference		Reference	
HR+/HER2+	0.47 (0.28–0.79)	0.004	0.62 (0.37–1.05)	0.074
HR‐/HER2+	0.86 (0.56–1.32)	0.486	1.13 (0.73–1.75)	0.585
HR‐/HER2‐	1.32 (0.84–2.08)	0.228	1.42 (0.90–2.25)	0.128
N		0.054		/
N0/N1mi	Reference		/	
N1	1.91 (1.09–3.36)	0.024	/	/
N2	2.49 0.62–10.03)	0.200	/	/
N3	2.53 (0.35–18.06)	0.355	/	/
NX	2.90 (0.72–11.71)	0.134	/	/
Surgery		<0.0001		<0.0001
No/Unknown	Reference		Reference	
Yes	0.15 (0.09–0.25)	<0.0001	0.21 (0.12–0.36)	<0.0001
Radiotherapy		0.001		0.028
No/Unknown	Reference		Reference	
Yes	0.60 (0.44–0.82)	0.001	0.70 (0.52–0.96)	0.028
Chemotherapy		0.613		/
No/Unknown	Reference		/	
Yes	1.12 (0.72–1.73)	0.613	/	/

Abbreviations: HR, hormone receptor; HER2, human epidermal growth factor receptor 2.

### 3.3 Nomogram construction

**FIGURE 3:**
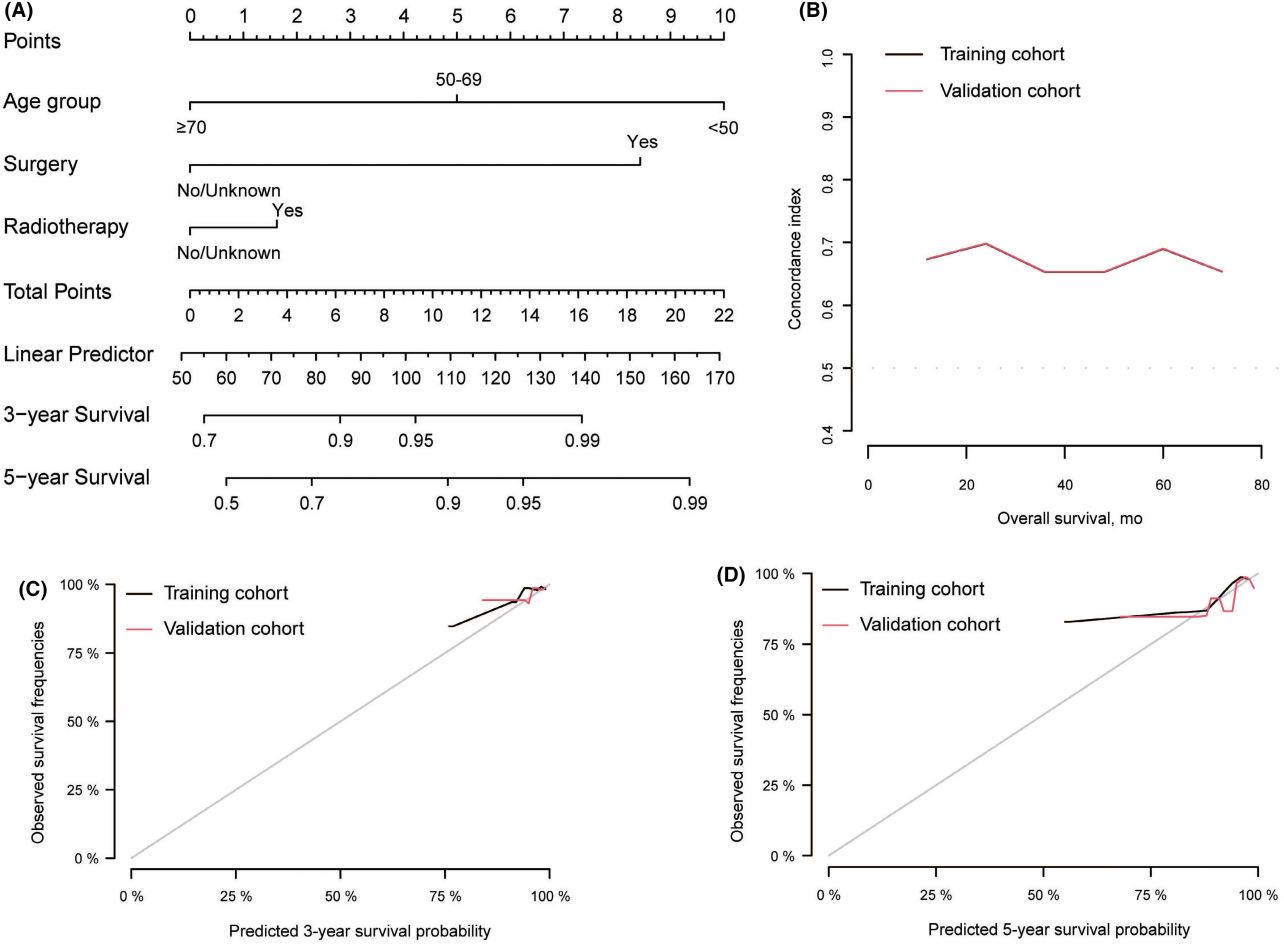
Construction and validation of nomogram. (A) Nomogram for predicting 3‐ and 5‐year overall survival of MIBC patients. (B) Concordance index curves of the nomogram in training and validation cohorts. (C) Calibration curves of the nomogram for 3‐year survival probability in training and validation cohorts. (D) Calibration curves of the nomogram for 5‐year survival probability in training and validation cohorts.

**FIGURE 4:**
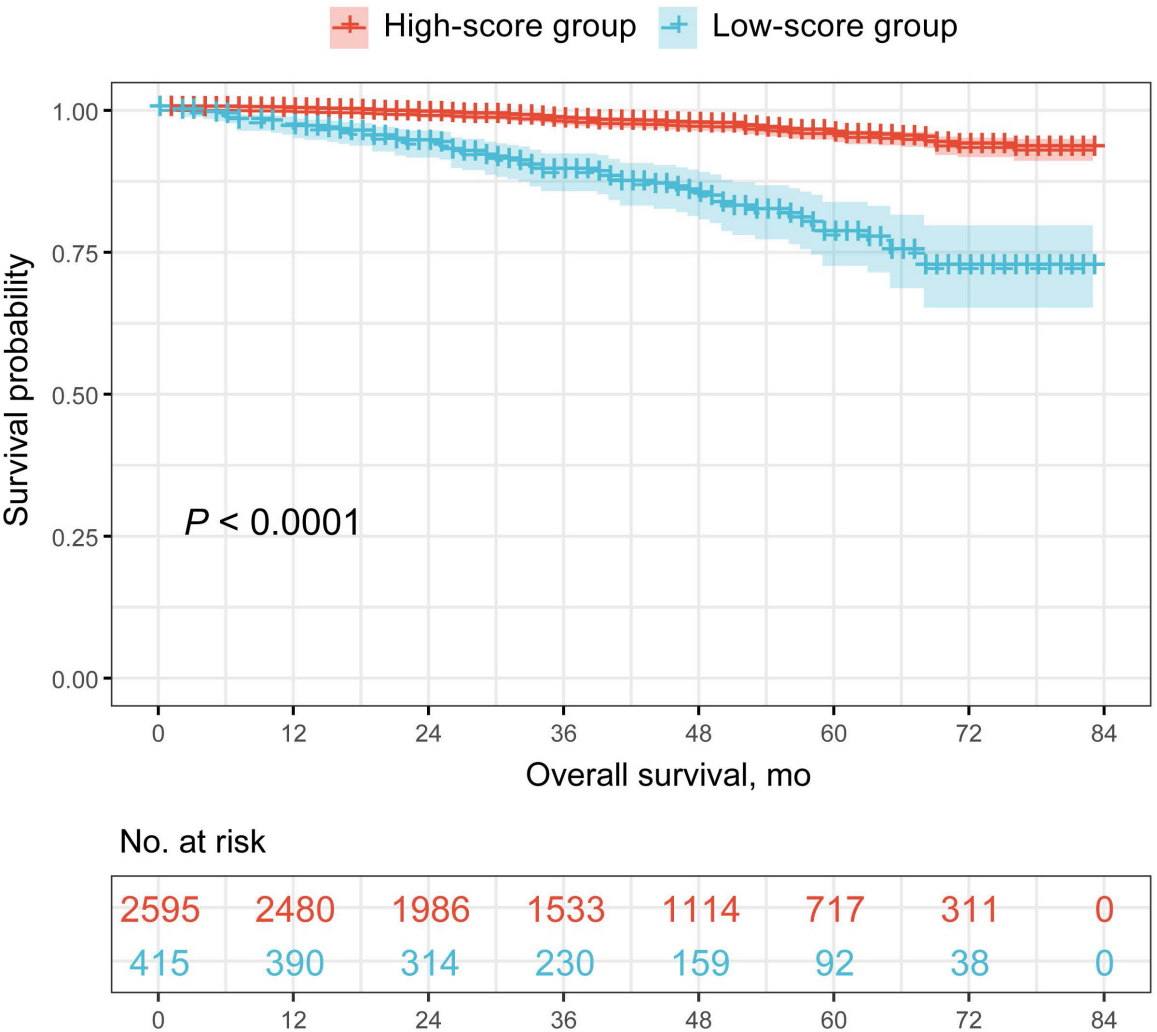
Survival curves stratified by score calculated in the nomogram for MIBC patients.

A nomogram was developed to predict 3‐ and 5‐year OS utilizing independent risk factors selected from the above‐mentioned multivariate regression analysis including age, surgery, and radiotherapy (Figure 3A). The C‐index of the training cohort and validation cohort were 0.73 (95% CI, 0.68–0.79) and 0.67 (95% CI, 0.58–0.77), respectively, indicating the prognostic model had decent discrimination (Figure 3B). The calibration curves of the two sets suggested that the model had favorable accuracy in predicting 3‐ and 5‐year OS of MIBC patients (Figure 3C and D). Then, all patients in the training cohort were divided into high‐score and low‐score group based on the total score acquired in the nomogram with a cutoff value as 9.99. The Kaplan–Meier curve showed a great difference in OS between high‐score and low‐score groups (*p* < 0.0001) (Figure 4).

## 4 DISCUSSION

Presenting as a special type of breast cancer, the understanding of MIBC is still insufficient. In this study, we elucidated the clinicopathological characteristics and prognosis of MIBC and originally explored the distinction among different molecular subtypes. We anticipate that our research into the above questions might provide clues to the future clinical practice.

Based on the status of HR and HER2, the eligible total population was divided into four groups. We made the comparison among the above four molecular subtypes and identified some differences. Demographically, the patients with HER2‐positive T1mic tumor were younger than HER2‐negative counterparts, in accordance with the previous study.^9^ Clinicopathologically, HR+/HER2‐ group manifested more as lobular carcinoma in regard to histologic type than other groups and tended to have more favorable pathologic characteristics. Invasive lobular carcinoma (ILC) is the second most common histologic type of breast cancer with a proportion of about 5–10%,^22^ which is believed to have unique clinical and pathological features compared with IDC, for instance, more common in older age, lower expression of HER2, and tending to be HR‐positive.^23^ However, microinvasive lobular carcinoma (MILC) is not fully elucidated due to its relative low incidence. Ross et al. summarized the clinicopathologic profile of 16 MILC cases from 1991 to 2009 and found that six patients whose HR or HER2 status could be obtained were all HR+/HER2‐, which is consistent with our study. Future investigation with more participants is recommended.^24^

In terms of treatment, almost all patients with T1mic tumor received surgery while only a small fraction of patients received chemotherapy (10.2%). Besides, slightly less patients received radiotherapy (44.9%). We further analyzed the different treatment choices among four molecular subtypes and found that patients in HR‐/HER2+ group were more likely to receive chemotherapy and less likely to receive radiotherapy compared with HR+/HER2‐ group. A recent study by Zhang et al. yielded similar results, though not statistically significant, which found patients with HER2+ MIBC had a higher trend toward chemotherapy compared with HER2‐ counterparts (29.4% vs. 8%).^10^ It was speculated that the more aggressive features of the HER2+ tumor and concern of the local recurrence caused the difference,^9^, ^10^ which could also be verified in our study.

We then focused on the prognosis of MIBC in the setting of different molecular subtypes and considerable distinction was detected. Subsequently, we made a pairwise comparison among them and it was of great interest to find that patients with HR+/HER2+ tumor had a favorable prognosis in both the whole MIBC population and MIBC patients without node macrometastasis, which was not the population that standard anti‐HER2 therapy was recommended for. Further research confirmed the above results, signifying that HER2 positivity in MIBC indicated better survival regardless of HR status. Previous studies showed that HER2 positive rate in MIBC was significantly higher than DCIS with a range from 36.5% to 61.8%.^9^, ^10^, ^15^, ^16^, ^25^, ^26^, ^27^ Our present data was relative lower with a rate of 31.5%. Although HER2 protein overexpression and/or HER2 gene amplification was believed to be related to a more aggressive clinical course,^28^, ^29^ it was not the case in our study. Similarly, several studies showed that HER2 positivity was not associated with lymph node metastasis or disease recurrence in MIBC.^10^, ^18^, ^25^, ^26^ Conversely, Fang et al. reported HER2 positivity was an indicator of worse disease‐free survival in DCISM patients free from adjuvant chemotherapy and trastuzumab.^30^ But in our view, HER2 positivity was not related to a worse OS, to say the least. Moreover, in T1micN0/N1mi patients, we have particularly noted that the difference in the OS between HR+/HER2‐ and HR‐/HER2‐ groups, and between HR+/HER2+ and HR‐/HER2+ groups reached statistical significance, respectively. In MIBC patients without lymph node involvement or with micrometastasis, HR positivity indicated a better prognosis regardless of the HER2 status, which did not apply for the whole MIBC population in the present study. MIBC patients with lymph node macrometastasis usually received more aggressive treatment than those without macrometastasis. We speculated that the survival reduction caused by HR negativity could be compensated by early and effective treatment. On the other hand, the prognosis of triple‐negative breast cancer (TNBC) tended to be worse than other molecular subtypes in N0/N1mi MIBC patients. However, in the whole MIBC population, the difference turned to be not significant. It was reasonable that unlike invasive breast cancer, the clinical presentations of TN subtype MIBC were not alike aggressive and might not be a surrogate indicator for the prognosis. We recommend to administrate intensive treatment to TN subtype MIBC patients with macrometastasis in order to prolong the survival.

Next, we compared the overall survival between T1mic and T1a. Data showed that there was no significant difference in prognosis neither in the whole population nor in any of the four molecular subtypes. The results remained the same when PSM was employed. A recent retrospective analysis conducted by Zheng et al. came to similar conclusions as we did, which reported that no difference in PFS, OS, or 5‐year mortality between T1mic and T1a.^15^ Other earlier studies by Fang et al.^30^ and Champion et al.^31^ also revealed that patients with T1mic displayed a comparable OS to those with T1a. Another study based on the SEER database showed the risk of breast cancer mortality for T1mic resembled small invasive cancers (0.2–1.0 cm). However, the risk was doubled when comparing T1mic with pure DCIS (*p* < 0.0001),^32^ which was also confirmed by Wang et al.^16^ All in a word, current evidence indicated that the clinical outcome of T1mic was more like T1a than Tis. Besides, the similarity of the OS did not change by any kind of treatment.

To date, divergence exists with respect to the appropriate treatment to T1mic. We investigated the effect of different treatment regimens on prognosis in this research. Interestingly, data showed that MIBC patients with a HR‐positive status who received radiotherapy tended to have a prolonged OS despite the HER2 status. We drew the same conclusion in T1micN0/N1mi patients. There was no related literature about radiotherapy in MIBC patients hitherto. To some extent, we could use DCIS and early‐stage breast cancer for reference. For DCIS, breast‐conserving surgery (BCS) with adjuvant radiotherapy is a standard approach and widely accepted.^33^ Several prospective randomized trials confirmed that the local recurrence rate was significantly reduced by half^34^ while the OS remained identical with the complement of radiotherapy.^35^ A recent published article further indicated that BCS plus radiotherapy was also a feasible choice for DCISM patients since there was no significant difference on local‐regional‐free survival (LRFS), distant metastasis‐free survival (DMFS), and OS between BCS plus radiotherapy (*n* = 74) and mastectomy only (*n* = 221) group.^36^ Here, we found additional OS benefit in T1mic by receiving radiotherapy, but only in HR‐positive population. Latest research by Wei et al. indicated that the omission of radiotherapy was associated with the adherence to endocrine therapy, for instance, patients who underwent radiotherapy were more likely to initiate endocrine therapy.^37^ Moreover, early discontinuation and nonadherence to adjuvant endocrine therapy predicted a decreased OS in early‐stage breast cancer patients.^38^ Notably, Jhawar et al. reported that ER‐positive early‐stage breast cancer patients (age ≥ 65 years) receiving adjuvant radiotherapy alone tended to have improved OS compared with endocrine therapy alone.^39^ In conclusion, we believed that the synergistic effect of both radiotherapy and endocrine therapy resulted in the favorable OS of HR‐positive patients. Based on our current outcome, radiotherapy was recommended for HR‐positive MIBC patients in favor of survival benefits. For HR‐negative population, the application of radiotherapy remains to be discussed with deliberation. As for adjuvant chemotherapy, MIBC patients regardless of molecular subtypes could not benefit from it with respect to OS. Similarly, some retrospective studies supported that adjuvant chemotherapy could not be advantageous regarding overall prognosis or 5‐year DFS.^13^, ^40^, ^41^ More than that, chemotherapy was found to be associated with inferior prognosis in LN‐negative patients.^40^ Plus, though not reaching significant difference, OS was decreased in the chemotherapy group compared with the non‐chemotherapy group (100% vs. 97.5%). Some previous studies indicated that MIBC patients with particular risk factors such as positive LN^42^ and negative HR^13^ could benefit from chemotherapy. However, these studies were usually based on a relatively small sample size (about 100 patients), and the estimated absolute survival benefit was rather little.^42^ The adverse effect brought from the adjuvant chemotherapy might be greater than the survival benefit. Thus, it is important to weigh the costs and effects when practicing chemotherapy.

We also discerned the predictive factors associated with the prognosis of MIBC. In the univariate model, age, ethnicity, molecular subtype, surgery, and radiotherapy were related to OS. These variables were included in the further multivariable analysis and results showed that age, ethnicity, surgery, and radiotherapy were independent prognostic factors. To be specific, old age (≥70 years), black ethnicity indicated a worse prognosis. Possible explanations were as follows. The comorbidities, undertreatment, and noncompliance result in the bad clinical outcome of elder patients. Biologic and genetic differences in tumors, and prevalence of risk factors might lead to the high mortality and low survival rates of black patients.^43^ The adoption of surgery, and radiotherapy indicated a better prognosis, in line with previous study.^16^ As last, to better quantify the prediction of survival, we developed a nomogram with satisfactory discriminant power. Influencing factors included age, surgery, and radiotherapy were utilized to build the prognostic model. The nomogram indicated that age had the greatest effect on the 3‐ and 5‐year OS, followed by surgery and radiotherapy. On this basis, a risk score stratification system was built. High‐score group had significantly superior OS compared with low‐score group, which indicated that more radical treatment might be necessary for the low‐score group. From this prospective, we provided a promising tool for physicians to better introduce the individual‐based therapeutics, instead of the insufficient basis of the receptor status or pathological stage.^44^ A recent study by Zhang et al. also constructed a nomogram utilizing the SEER database to predict the breast cancer‐specific survival (BCSS) for MIBC patients.^45^ But, the wide time span of patients' enrollment and lacking of clear information regarding molecular subtype might partially affect the reliability of the prognostic model.

The present study should be explained with consideration since there exist several limitations. For starters, this research is a retrospective analysis based on the SEER database, having some inevitable bias compared with prospective study. The bias could not be fully balanced although the PSM was conducted to reduce them as much as possible. And the quality and missingness of the data varies across the SEER database. Secondly, it should be pointed out that making correct diagnosis of T1mic tumor is not easy in terms of the pathological approach. The small invasive component of the sample might affect the pathological diagnosis, thus confounding the ultimate outcome of this analysis. Thirdly, the prognostic indicator we used herein was OS rather than BCSS, which may affect the results. In addition, treatment information about the endocrine therapy and targeted therapy were not recorded in this database, so it was quite difficult to access the panorama of the prognosis of MIBC patients divided into different molecular subtypes. Also, other important clinical parameters like Ki‐67 and lymph vascular invasion were not available as well. Next, we will conduct further analysis utilizing data from the National Cancer Center to consolidate the present evidence.

## ACKNOWLEDGMENTS

The authors did not receive financial support for the research, authorship, and publication of this article.

## CONFLICT OF INTEREST

The authors have no conflicts of interest to declare.

## AUTHOR CONTRIBUTIONS

YH conceived the study and collected the data. HX and YH analyzed and interpreted the data. HX wrote the manuscript. YH, YuW, and YaW revised the manuscript. BX and JW supervised the study. All authors contributed to the article and approved the submitted version.

## ETHICS STATEMENT

Not applicable.

## Supporting information


Appendix S1

## Data Availability

The data used and/or analyzed during the current study are available in the Surveillance, Epidemiology, and End Results database.
