# Supplementary material for: Clinicopathological characteristics and prognosis of microinvasive breast cancer: A population‐based analysis
Source: Cancer Med. 2022 May 22;11(23):4501–12. doi: 10.1002/cam4.4839 (PMC9741986; doi:10.1002/cam4.4839)

Supplementary Materials

TableS1. Pairwise comparisons of OS between different molecular subtypes in MIBC patients.

| HR+HER2- 79.32 (78.69-79.95) |  |  |  |
| --- | --- | --- | --- |
| 0.003 | HR+HER2+ 81.28 (80.45-82.11) |  |  |
| 0.487 | 0.054 | HR-HER2+ 79.72 (78.49-80.95) |  |
| 0.224 | 0.001 | 0.146 | HR-HER2- 78.19 (76.29-80.10) |

TableS2. Pairwise comparisons of OS between different molecular subtypes in T1micN0/N1mi patients.

| HR+HER2- 79.53 (79.31-79.74) |  |  |  |
| --- | --- | --- | --- |
| *P*<0.001 | HR+HER2+ 80.80 (80.31-81.30) |  |  |
| 0.634 | 0.015 | HR-HER2+ 79.69 (78.91-80.47) |  |
| 0.019 | *P*<0.001 | 0.054 | HR-HER2- 78.60 (77.78-79.43) |

FigureS1. The comparison of OS between different ER, PgR, HR and HER2 status ER=Estrogen Receptor, PgR=Progesterone Receptor, HER2=Human Epidermal Growth Factor Receptor 2


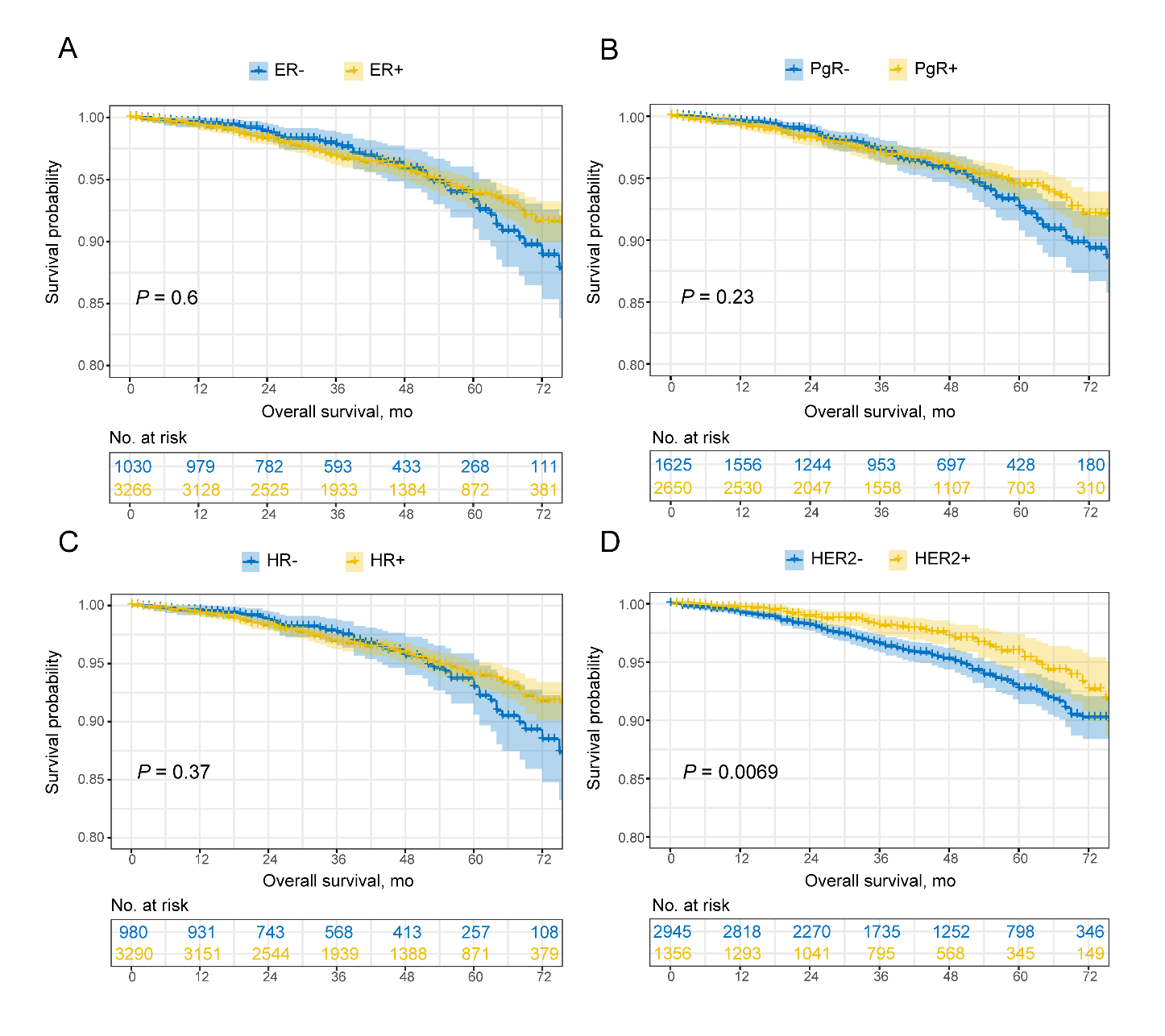


FigureS2. The comparison of OS between T1mic and T1a patients.


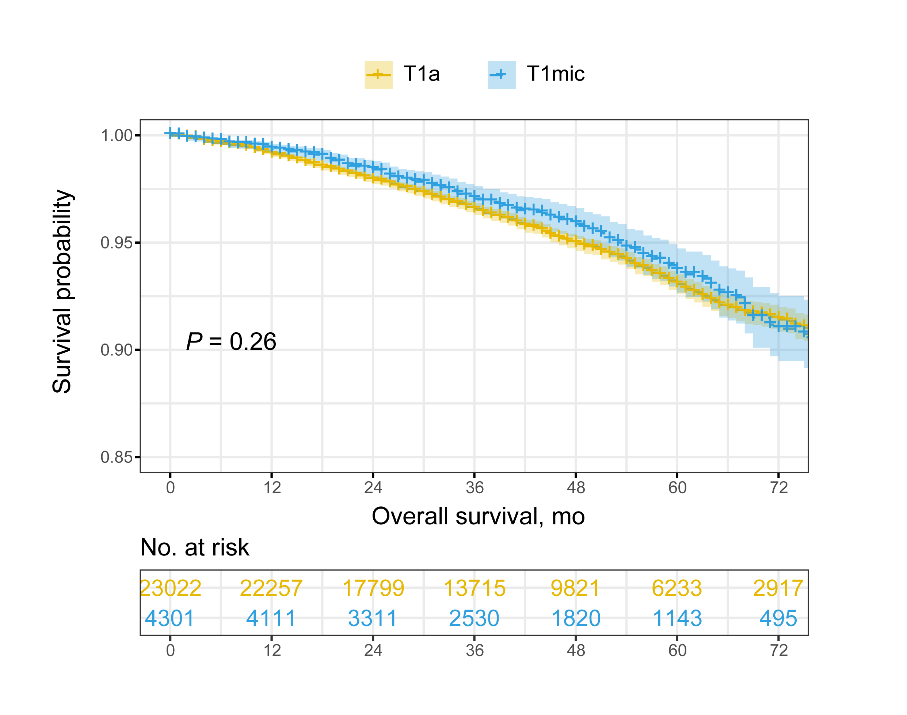


FigureS3. The comparison of OS between T1mic and T1a patients in different subtypes.


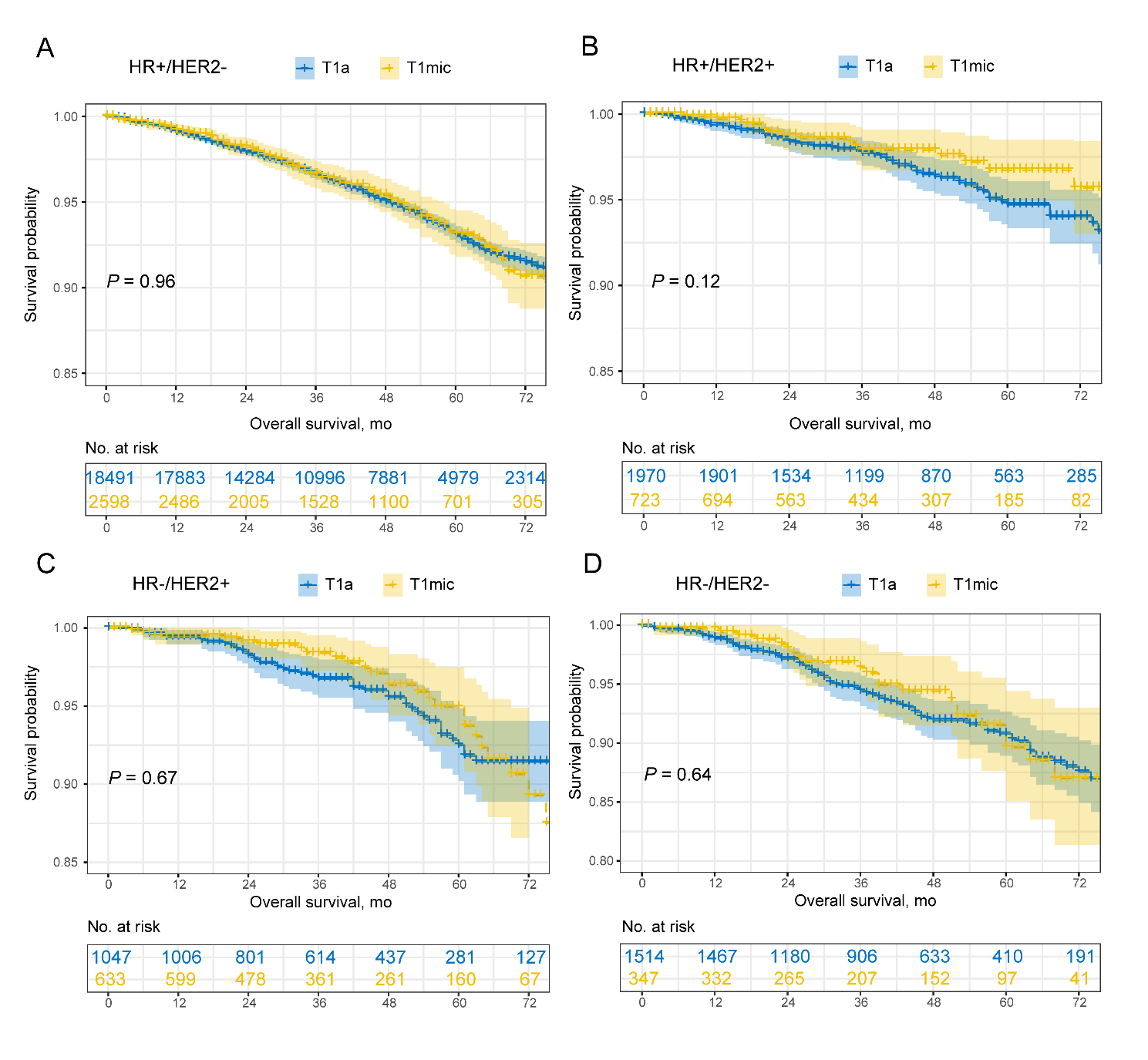


FigureS4. The comparison of OS between T1mic and T1a patients when controlling factors at baseline in a 1:2 ratio.


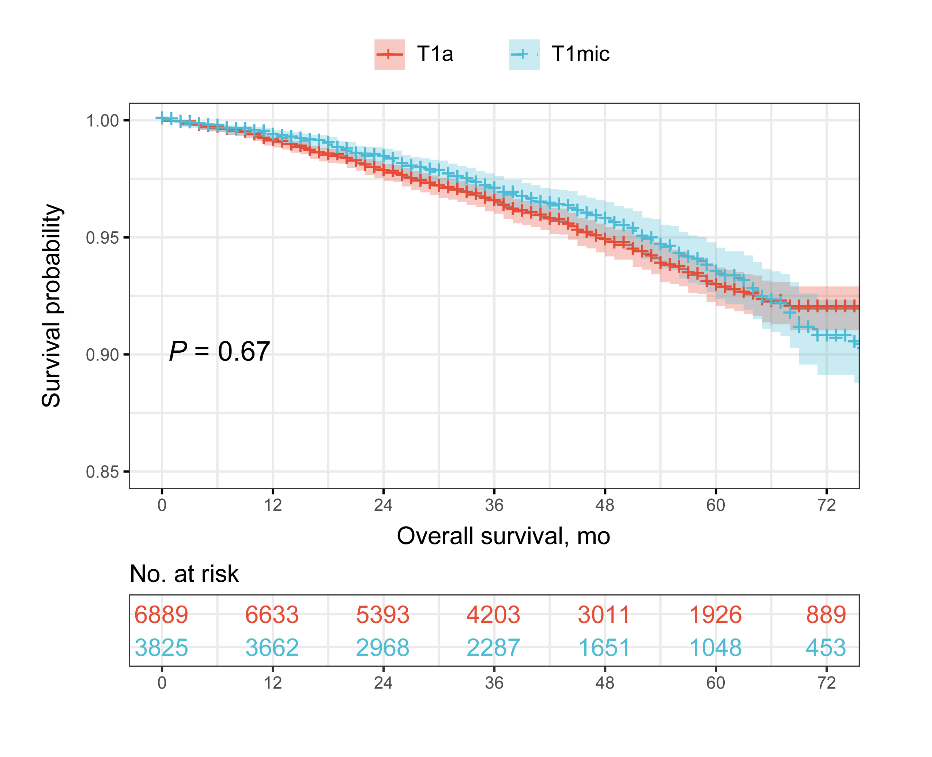


FigureS5. The comparison of OS between T1mic and T1a patients in different subtypes when controlling factors at baseline in a 1:2 ratio.


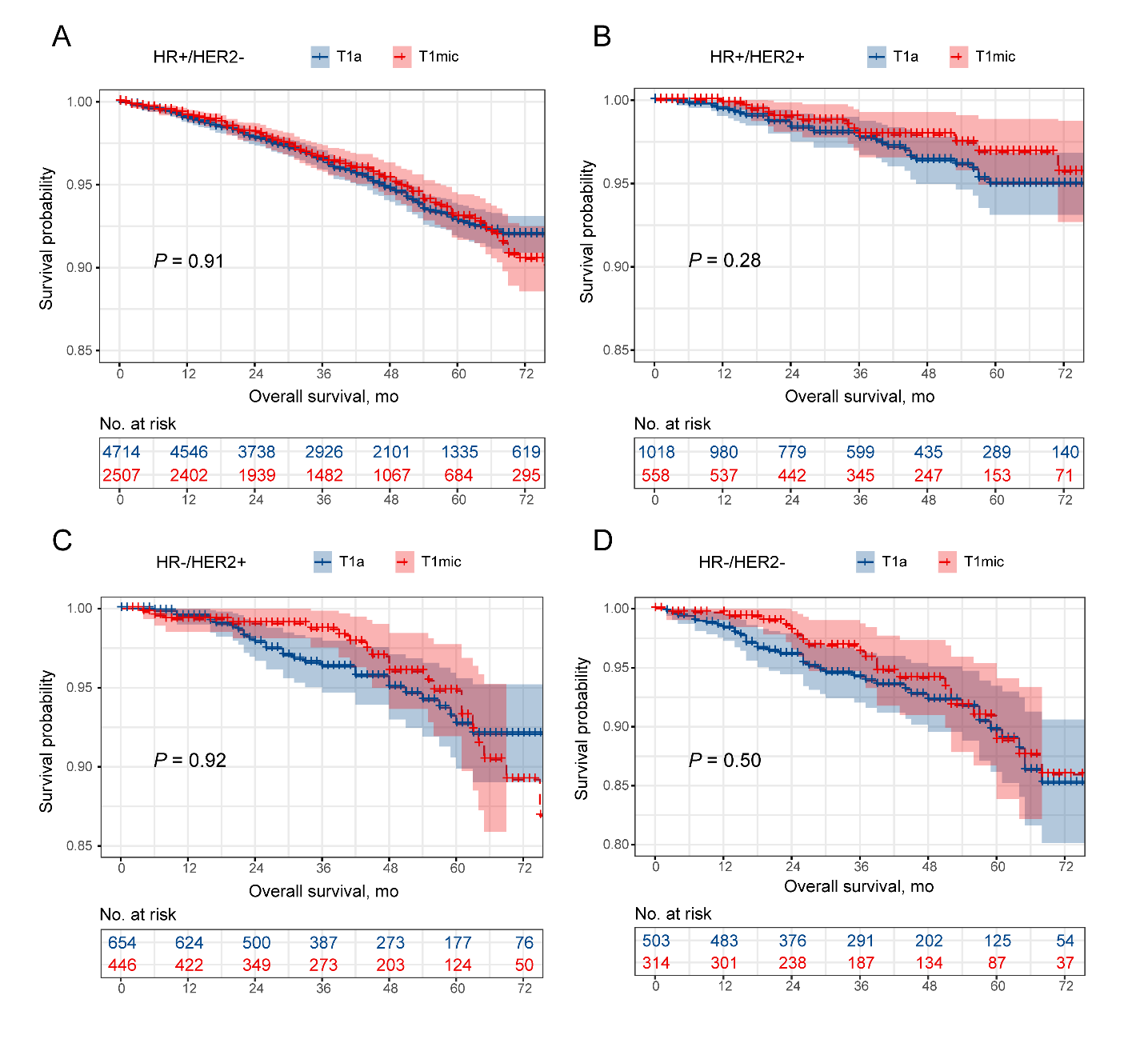


FigureS6. The impact of radiotherapy on OS in different subtypes


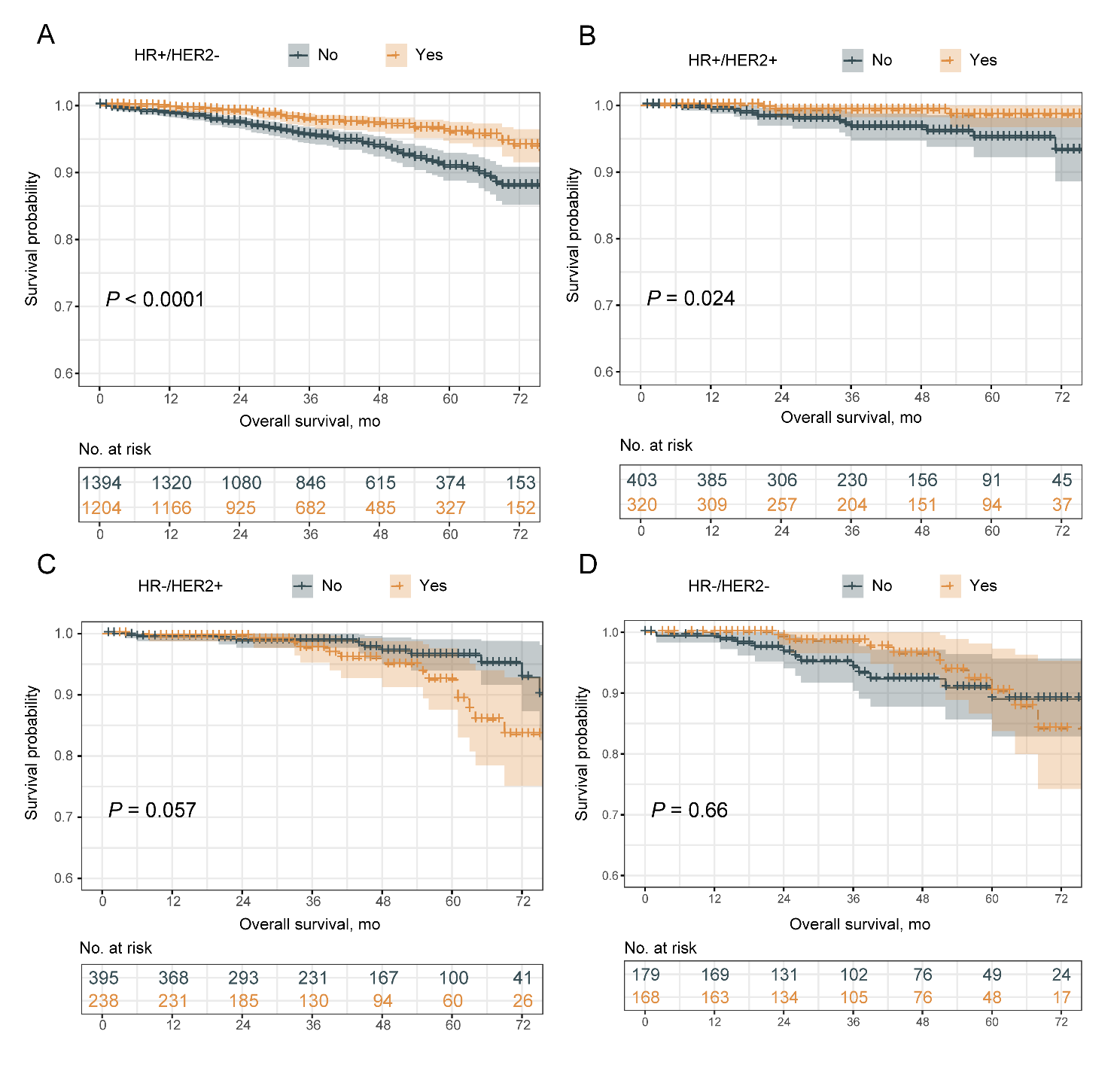


FigureS7. The impact of chemotherapy on OS in different subtypes


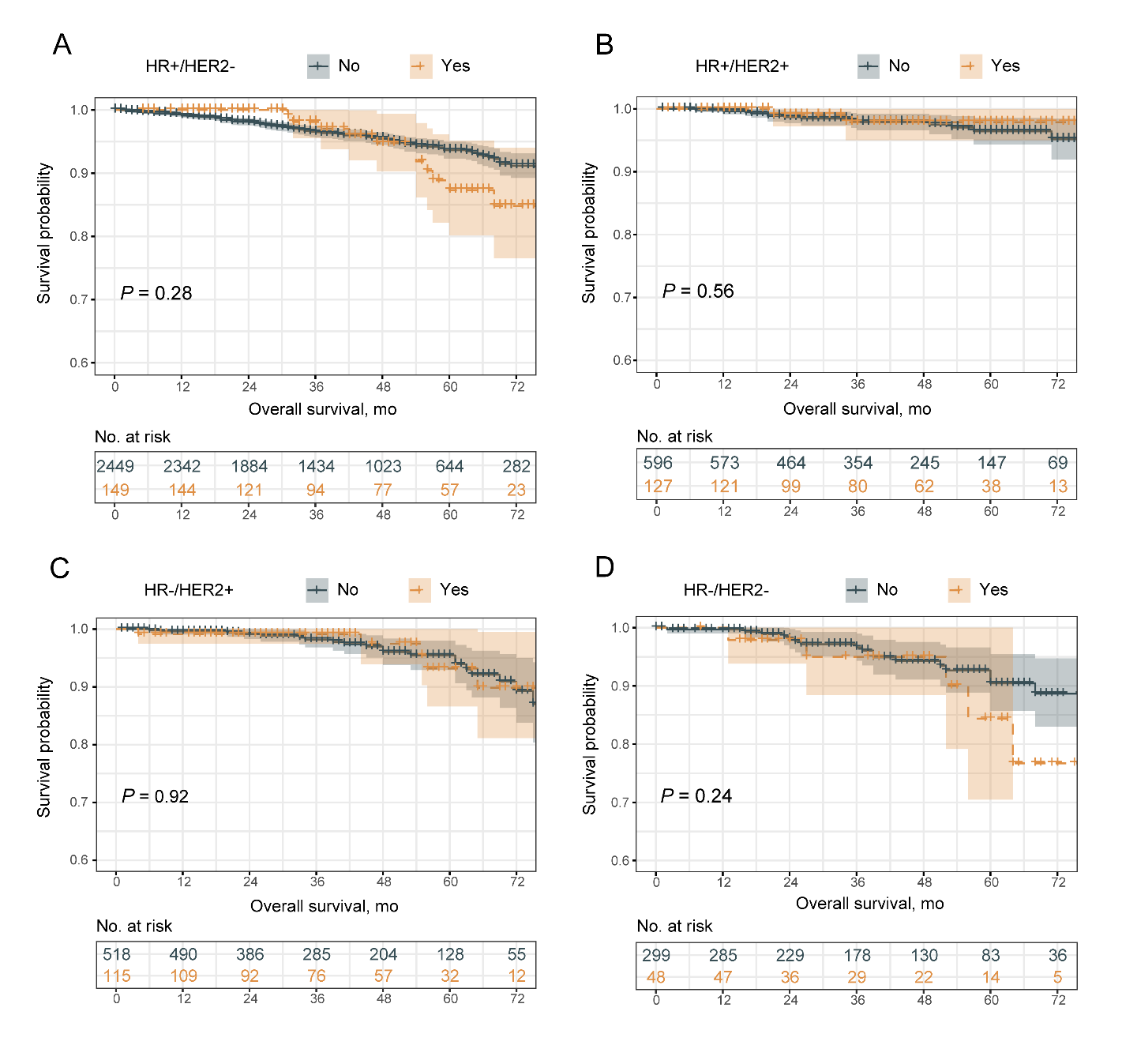


FigureS8. The impact of radiotherapy on OS in different subtypes among T1micN0/N1mi group


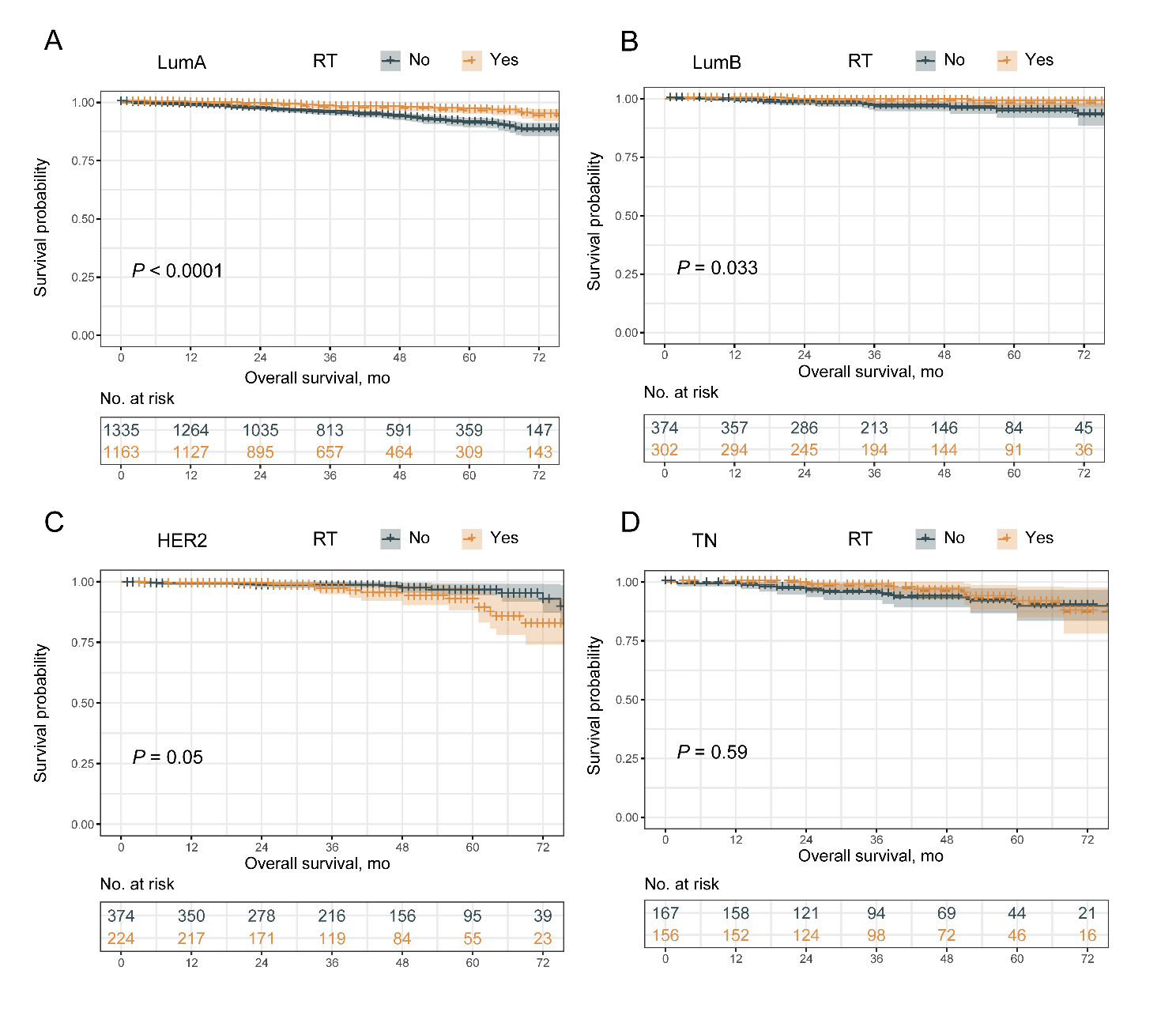


RT=radiotherapy

FigureS9. The impact of chemotherapy on OS in different subtypes among T1micN0/N1mi group


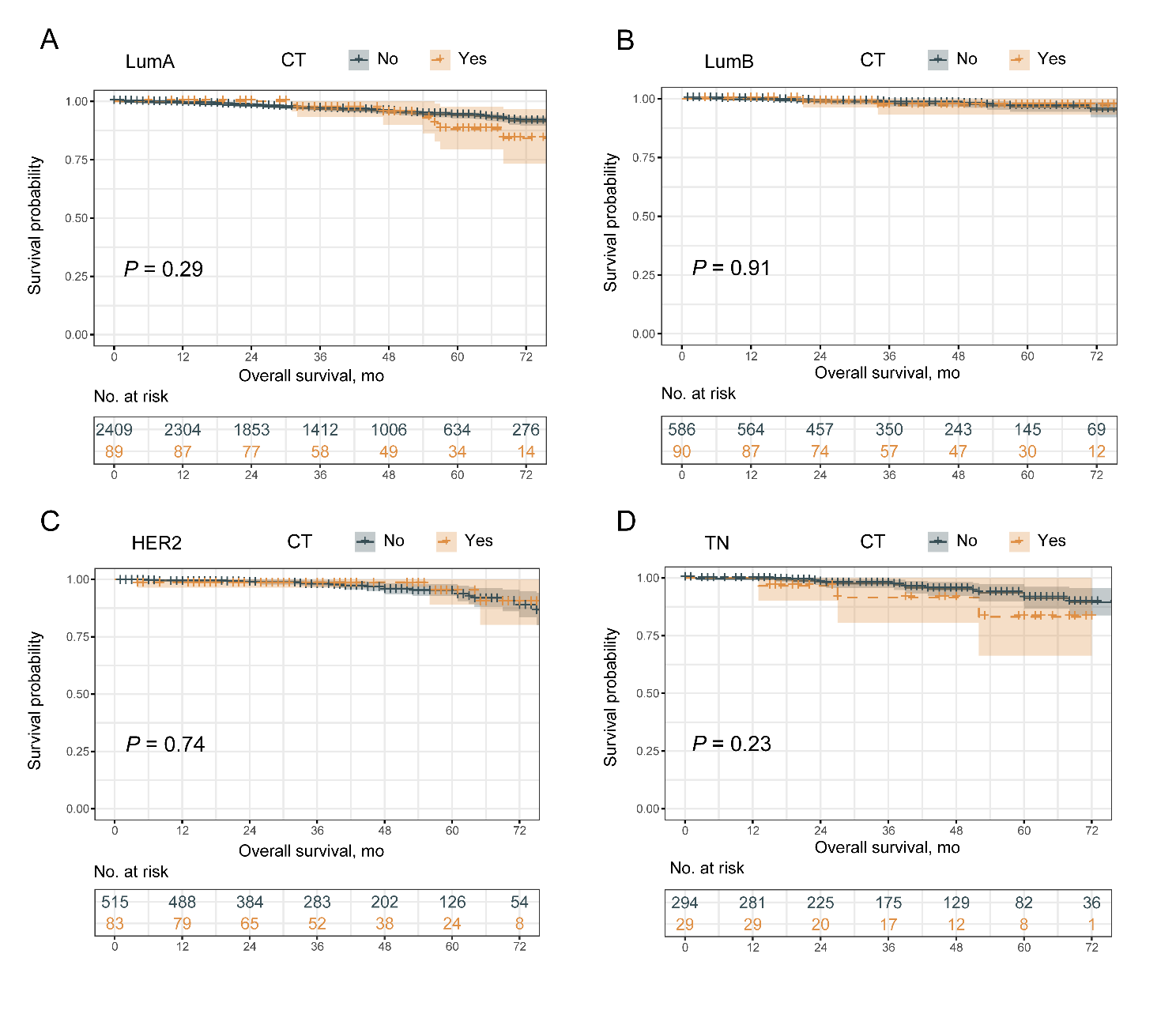


CT=chemotherapy

FigureS10. The differences of OS between T1mic and T1a patients after receiving surgery (A and B), radiotherapy (C and D), or chemotherapy (E and F). (Figure B, D and F control factors at baseline in a 1:2 ratio)


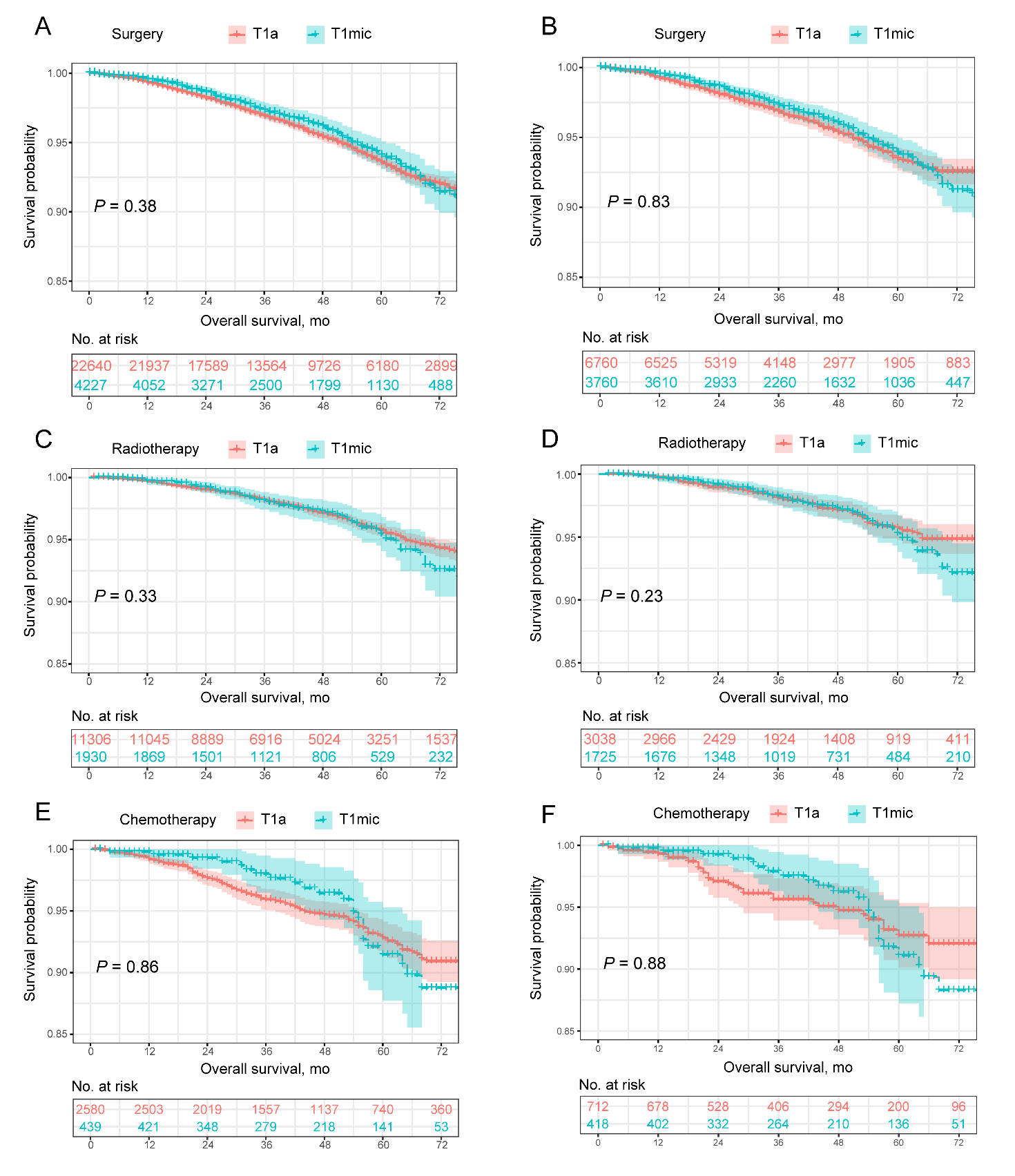

Supplement: Supplementary file 1 — Appendix S1 [file CAM4-11-4501-s001.docx]
